# Microbes as Teachers: Rethinking Knowledge in the Anthropocene

**DOI:** 10.1111/1751-7915.70195

**Published:** 2025-07-14

**Authors:** Rachel Armstrong

**Affiliations:** ^1^ Professor of Design Driven Construction for Regenerative Architecture, Department of Architecture, Faculty of Architecture Brussels Belgium

**Keywords:** anthropocentrism, epistemology, learning, literacy, microbes, microbiome, symbiosis

## Abstract

This opinion piece proposes that the environmental crises of our time arise from a failure to recognise the vital role of microbes in sustaining life on Earth, where ecosystems have been shaped for billions of years by microbial processes, including oxygen production, nutrient cycling and climate regulation. Yet the idea that microbes can ‘teach’ us how to navigate complexity, adapt across scales, and sustain planetary systems is still marginalised in science, policy, and education. A paradigm shift is proposed: microbes must be reframed as active collaborators in solving global challenges. This perspective is grounded in microbial ecology, Indigenous knowledge, and ethical philosophy, advocating for ‘learning’ through and with microbial life. To institutionalise this transition, policy and educational reforms are urged, centring microbial literacy as a foundation for ecological understanding. By integrating microbial agency into human knowledge systems, societal actions could be realigned with the biochemical and evolutionary logics that have sustained life for millennia. Ultimately, a deeper engagement with microbial knowledge is called for—one that informs a more sustainable future.

## Graphical abstract

This essay positions microbes as active ‘teachers’ of ecological wisdom. Microbes demonstrate resilience, symbiosis and ecosystem intelligence through their metabolic networks, providing a dialogic learning experience. Emerging technologies enable reciprocal engagement with microbes, repositioning humans from observers to embedded learners of the vital 3.8‐billion‐year microbial legacy, offering lessons for bioremediation, decentralised governance, and post‐anthropocentric ethics. Key insights and actionable strategies for embracing microbes as ‘teachers’ are presented, aligning with the Initiative for Microbial Literacy (IMILI) principles. Microbial wisdom—collaboration, creativity, adaptation, and symbiosis—are presented as a blueprint for rethinking education, policy, and our relationship with life's unseen majority.

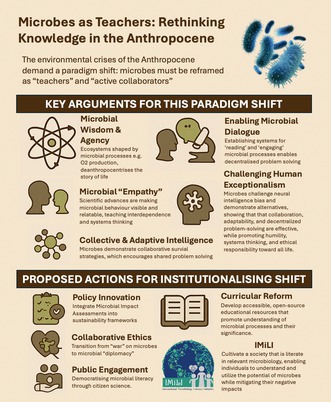

## 1 The Anthropocene as a Crisis of Perception

The crises of the Anthropocene—climate collapse, pandemics, and biodiversity loss—demand a fundamental rethinking of humanity's relationship with nature (Crutzen and Stoermer 2000; Steffen et al. 2007). Microbes, with their 3.5‐billion‐year evolutionary history, offer critical lessons in adaptation (Vicuña and González 2021), from horizontal gene transfer (Brochier‐Armanet and Moreira 2014) to the collective intelligence of biofilms (Gupta et al. 2016). These microbial strategies form an *epistemic tradition*—a way of knowing or understanding the world—which has been made visible through advances in genomics, live‐cell imaging, and bioinformatics. By translating microbial communication and metabolic innovation into data, we gain access to evolutionary strategies that are urgently needed in this era of planetary stress. Yet despite their relevance, microbial perspectives remain marginalised in policy and education, which continue to prioritise anthropocentric models of resource use (Armstrong 2025). This opinion piece repositions microbes as “teachers”—active mentors whose evolutionary intelligence can form new approaches to circularity, adaptation, and resilience, potentially reshaping the future of sustainable innovation (Hughes and Armstrong 2020). In doing so, it also responds to the call for Societally Relevant Microbiology Education and Outreach as championed by the International Microbiology Literacy Initiative (IMiLI), which aims to enhance global microbiology education by developing freely accessible, learner‐centric resources that promote critical and systems thinking (Timmis et al. 2024).

## 2 Microbial Empathy: Walking in Unfamiliar Skins

Empathy, as Harper Lee wrote, means imagining life from another's perspective—“walking around in another's skin” (Lee 2020, 16). Indigenous cosmologies have long recognised non‐human personhood, offering frameworks for respectful coexistence (Dussart and Poirier 2023). This perspective extends to microbes, which actively shape Earth's systems through intelligent, agent‐like behaviour (Smith 2023). Microbial empathy challenges industrial models of extraction by acknowledging that organisms like algae and fungi follow their own rhythms and form life‐sustaining networks (Simard et al. 1997). True collaboration requires aligning with these natural timelines—minutes for bacteria, days for fungi—rather than imposing human‐centred schedules (Rousk and Bååth 2011). Ethically, we must ask: who benefits? Does a bioreactor enrich *Spirulina*'s existence, or merely improve human air quality? When microbes have access to nutrients and a healthy environment, they become more effective at cleaning pollution and storing carbon. This reveals a biotechnological imperative: microbial well‐being is essential to ecosystem health. It also echoes rights‐for‐nature debates, framing microbial care as both a scientific and ethical responsibility (Wesche 2021). From this perspective, microbial empathy reframes success not as domination, but as coexistence—where algal vitality and fungal robustness become new indicators of ecological health. Aesthetic values change as well: algal tanks and walls constructed from fungal building blocks redefine beauty in terms of metabolic vitality rather than human preference (Armstrong 2022). Supporting microbial life, then, becomes both a practical strategy and a philosophical commitment to living well together on a shared planet.

## 3 Nature as Teacher: Metaphor vs. Literal Agency

The Enlightenment cast nature as a passive resource—an idea that still shapes how we treat the microbial world. Even in this unfolding Age of Biology (Hockfield 2019), as microbial strategies like horizontal gene transfer inspire circular economic models (Mostafa et al. 2024), microbes are often regarded as primitive, their sophisticated, adaptive, decision‐making capacities overlooked (de Marques Sá and Viana 2023). This reinforces an extractive mindset, where microbes are used but their autonomy is not recognised. In contrast, animist and new materialist philosophies (Barad 2007; Bennett 2010; Benson 2019) reimagine nature as an active teacher. From this perspective, learning from microbes requires more than observation—it demands an *ethics of receptivity*—that is, we must set aside anthropocentric assumptions and recognise microbial behaviours as real, impactful actions. Methanotrophs reduce atmospheric methane (Guerrero‐Cruz et al. 2021), and *Geobacter* species purify water through electrogenic respiration (Tejedor‐Sanz, Fernández‐Labrador, et al. 2018; Tejedor‐Sanz, Li, et al. 2018)—these are acts of planetary care, not metaphors. Metaphorical pedagogy keeps microbes at a distance; literal pedagogy invites us to listen. As climate collapse accelerates, microbes are already responding. The real question is: are we ready to learn? (Hughes and Armstrong 2020).

## 4 Seeing the Unseen: Technologies for Microbial Dialogue

Individual microbes are imperceptible to the naked eye but when encountered *en masse* they are unsightly—as slime, scum or symptoms of dysbiosis. This perceptual limitation, shaped by human evolution to prioritise large‐scale, visible forms—what Myra Hird calls ‘big like us’ (Hird 2009, 2)—poses a fundamental challenge: how can we meaningfully engage with microbial life? The answer lies in technology. Over the past 300 years, a succession of scientific tools has enabled new modes of microbial encounter. Each innovation expanded our capacity to observe microbes, shaped the kinds of questions we can ask—and framed the lessons we can learn. Technologies do not merely reveal microbes; they structure the terms of our dialogue with them. This journey began with microscopy, from Leeuwenhoek's first glimpse of ‘animalcules’ (Lane 2015) to the near‐atomic resolution of cryo‐electron microscopy (Razi et al. 2017). Genomic tools followed: Sanger sequencing enabled detailed analysis of microbial DNA (Sanger and Coulson 1975; Sanger et al. 1977), while metagenomics enabled the diversity and function of microbial communities in different habitats to be studied (Handelsman et al. 1998). Live‐cell imaging marked a turning point, making microbial behaviour visible in real time (Frigault et al. 2009). Fluorescent protein tags help visualise quorum‐sensing interactions in real time (Welsh and Blackwell 2016), while CRISPR‐based reporters enable dynamic tracking of gene expression (Hsu et al. 2013). Tools from synthetic biology, such as CRISPR‐Cas9, allow precise genome editing (Jinek et al. 2012). Together, these technologies reveal how microbes communicate—through chemotaxis, quorum sensing, and metabolite exchange—demonstrating their diverse strategies for sensing and interacting with their environment. These behaviours reflect microbial “thinking”: chemotaxis as problem‐solving (Long et al. 2017), epigenetic memory as adaptation (Lambert and Kussell 2014), and horizontal gene transfer as collaborative innovation (Margulis and Sagan 2002; Jain et al. 2003). To engage in true dialogue with microbes, we must attune ourselves to their timescales, sensory modalities, and metabolic languages—transitioning from anthropocentric monologue to genuine interspecies conversation.

## 5 Disruption as Lesson: The Microbial Turn and Its Shadows

In the early 21st century, microbes were increasingly reframed as collaborators (Paxson and Helmreich 2014), inspiring models of decentralised networks (Hird 2009) and biofilm‐based infrastructure (Cabanek et al. 2020; Sádaba et al. 2025). This ‘microbial turn’ has positioned microbes as teachers of resilience, circularity and ecological intelligence, but this celebratory framing requires nuance. While many microbes sustain ecosystems, others—such as *Yersinia pestis* (Bramanti et al. 2016) and *Plasmodium* (Belachew 2018)—remind us that microbial adaptability is amoral, capable of driving both symbiosis and pathogenesis depending on the context. This complexity echoes Carl Woese's foundational insights: microbial life is diverse, evolution is networked, and microbial function is relational—shaped by context, cooperation, and competition (Woese and Fox 1977; Woese et al. 1990; Woese 2004). Microbes do not operate in isolation. They are deeply embedded in the living world, constantly interacting with their environments and with one another. This challenges traditional hierarchies and calls for a new way of thinking. To genuinely collaborate with microbes—active agents in vast, interdependent ecosystems—we must adopt a systems perspective that recognises their roles, relationships, and responses within broader ecological networks. Their potential emerges through cooperation, not control. Examples like CRISPR immunity, deep‐sea nutrient cycling, and quorum‐sensing show how microbial intelligence is collective and adaptive. Systems science offers the tools to understand this complexity. It emphasises feedback loops, emergent behaviour, and relational thinking over linear causality. But science alone is not enough. We also need intellectual openness—to perceive microbes as embedded agents, shaping and shaped by the living world. This transition has real‐world implications. It means designing interventions that work with microbial rhythms: combining phage therapies with vaccines, replacing chemical agriculture with probiotics, and treating microbial health as essential to planetary health. Alongside rapid scientific and technological advances, we need a broader cultural and educational transformation—one that asks: if microbes are already shaping our world, what more might we discover by allowing them to shape how we learn?

## 6 Toward Microbial Classrooms

The microbial world demands an ambitious reimagining of education—not in literal classrooms, but as a paradigm shift in how we institutionalise knowledge about life's foundational actors. This transformation requires three interdependent pillars: curricular reform, public engagement, and policy innovation—all recognising microbes as co‐teachers rather than passive subjects.

### 6.1 Curricular Foundations: Rewriting the Textbook

Transforming biology education requires dismantling its anthropocentric foundations, where microbes appear as marginal notes rather than central narrators of life's story. This begins by recasting core concepts to foreground microbial agency: photosynthesis should be taught as cyanobacterial geoengineering—a 2.7‐billion‐year‐old innovation that oxygenated Earth's atmosphere, triggering the Great Oxidation Event and inventing the planet's primary energy‐capture system (Blankenship 2010). Similarly, carbon cycles must be reframed as a dynamic partnership between methanotrophs and phytoplankton, whose metabolic negotiations maintain atmospheric equilibrium at planetary scales (Falkowski et al. 2008). Such changes demand replacing the hierarchical ‘tree of life’ with Carl Woese's networked model of horizontal gene transfer, where microbial alliances—not isolated competition—drive evolutionary innovation. Curricula must also integrate this science with ethical reflection: contrasting 20th‐century medicine's ‘war on germs’ (and its legacy of antibiotic overuse and dysbiosis) with 21st‐century symbiosis frameworks that position human health as inseparable from microbiome stewardship. These reforms decentralise human exceptionalism, recognising microbial communities as the original and ongoing architects of Earth's operating systems.

### 6.2 Democratising Microbial Literacy

Democratising microbial literacy requires moving beyond reductive narratives of pathogens and probiotics that dominate public discourse. Citizen science initiatives—from DIY smartphone microscopy to participatory biofilm art—make microbial worlds tangible to non‐specialists (Hester et al. 2018). These projects cultivate attentiveness to life at invisible scales. Broader impact requires policy frameworks that transform individual engagement into societal participation. For example, municipal wastewater treatment plants could host public microbiology labs, where citizens use PCR to monitor local microbial communities—linking metagenomic data to urban pollution policies or land‐use decisions that include microbial impact assessments (Fierer et al. 2021; Jurburg et al. 2022). Funding bodies should also support art‐science collaborations like the ALICE (Active Living Infrastructure: Controlled Environment) project at ZKM, Karlsruhe—an interactive installation that visualised microbial electroactivity in real time. Using microbial fuel cells, the system translated biofilm responses to stimuli into animations, enabling viewers to witness microbial behaviour and its relevance to wastewater processing (Armstrong et al. 2024). Such initiatives promote deeper understanding and reciprocal engagement between humans and microbial systems.

### 6.3 Policy Levers for Microbial Partnerships

For microbial stewardship to transition from theory into practice, policymakers must adopt frameworks that integrate microbial considerations into governance, industry, and public discourse. A foundational step is the introduction of *Microbial Impact Assessments* (MIAs), modelled on *Environmental Impact Assessments* but designed to evaluate how agricultural or urban development projects affect microbial communities—from soil archaea to phyllosphere bacteria. By establishing legal thresholds for microbiome disruption, such as declines in mycorrhizal network functionality or nitrogen‐fixing rhizobia, MIAs could ensure microbial health is considered alongside macroscale ecosystems in land‐use decisions.

The feasibility of such assessments has been unlocked by advances in microbiome analytics. Government agencies can now digitise and interpret the *global environmental microbiome*—the unique microbial fingerprints of every location on Earth—from farms to forests to factories (Zhang et al. 2024). A pioneering tool in this space, *Microbiome Geographic Population Structure* (mGPS), leverages artificial intelligence to analyse microbial communities and determine their geographic origin with remarkable precision (Zhang et al. 2024). By combining high‐throughput DNA sequencing, machine learning, and an expanding geospatial database of microbial data, mGPS transforms biological signatures into actionable insights. By evaluating the relative abundances of *Geographically Informative Taxa*, the system functions like a biological GPS. It can trace microorganisms found on everyday items—skin, cell phones, soil or water—with applications spanning forensic science, supply chain transparency, and ecological monitoring. For example, mGPS can verify the true origin of goods, such as detecting whether coffee beans labelled as Colombian originated in Ethiopia, based on the microbial communities associated with their processing environments. Similarly, in forensics, it can help reconstruct a suspect's recent movements from microbial traces they leave behind.

Earlier microbiome‐based geolocation methods were limited by small datasets, narrow ecological scope, and rigid classification models. While they could often identify a sample's origin at the city or national level, they struggled to generalise beyond predefined categories. The mGPS model advances this field by predicting geographic coordinates that are not in its training data–an essential step for real‐world forensic and ecological applications. Still, challenges persist. Microbial signatures are constantly reshaped by global human and material movement, complicating location inference. Targeted sampling at transit hubs like airports could help maintain accuracy. For mGPS to be truly effective, it must be paired with regularly updated urban microbiome maps and achieve near‐perfect precision.

## 7 Scaling up: Microbial Metrics in Global Frameworks

To institutionalise microbial stewardship, microbial metrics must be integrated into global sustainability frameworks. Just as deforestation and species loss are tracked under the UN Sustainable Development Goals (SDGs), microbial diversity—such as mycorrhizal richness per hectare—should be included as a measurable indicator of ecosystem health, particularly for carbon sequestration and soil fertility.

Attention is also needed for *material innovation*, as mycelium‐based composites and other biomaterials enter mainstream use. Standards must certify not only performance but also *ethical microbial sourcing*, requiring lifecycle analyses that weigh ecological benefits (e.g., carbon capture, biodegradability) against cultivation ethics–applied not only to novel biotechnologies but also to conventional processes, enabling meaningful comparisons and informed transitions. Here, microbial analytics could play a pivotal role by tracking community impacts across supply chains (Green et al. 2024).

## 8 Reframing Public Narratives

Structural policy changes must be accompanied by a change in public perception. Current advertising regulations, for instance, permit biocidal claims like ‘kills 99.9% of germs’—messages that reinforce adversarial relationships with microbial communities essential to human and planetary health (Gilbert et al. 2018). Instead, evidence‐based campaigns, akin to antibiotic stewardship efforts, could promote *microbiome‐aware practices* in household cleaning and urban planning. The goal is to promote *microbial citizenship*: a societal recognition of our interdependence with—and responsibility toward—the microbial networks that sustain all life.

## 9 Case Studies in Collaborative Ethics

The ethics of microbial engagement—which refers to the moral and philosophical considerations involved in how humans interact with microbial life—cannot be reduced to simple binaries of exploitation versus non‐intervention. Instead, it requires a spectrum of collaborative practices negotiated within specific contexts. Take the case of mycelium: in mycoremediation, its metabolic pathways are harnessed without disrupting its living networks. Yet in producing mycelium biocomposites, fungi are cultivated to bind organic waste materials—wood, straw, hemp, coffee grounds—into structural forms, only to be deliberately terminated through heat treatment to make them inert and safe for human use. This transition from living collaborator to inert material raises ethical questions. We must consider the ecological value of preserving fungal vitality against the practical necessities of safe human applications (Sharma et al. 2024). Material science could benefit from developing gradients of collaboration, preserving fungal life in applications like living filtration systems, while accepting controlled termination in others such as packaging and insulation. Lifecycle analyses could help evaluate trade‐offs, such as the carbon sequestration potential of living fungal networks versus the durability of mycelium‐based alternatives to a range of plastics (Armstrong 2021).

Bacterial communication systems offer another valuable model for considering our ethical relationship with microbes. Quorum sensing—the chemical language that coordinates bacterial behaviour—can, if disrupted, lead to unintended consequences such as increased virulence or ecological cascades (Bassler and Losick 2006). Recognising these systems as evolved networks of interaction demands biosafety protocols with rigour comparable to those used in research on human subjects. For instance, synthetic biologists engineering *Pseudomonas* for bioremediation might use microfluidic containment to minimise culturing artefacts or deploy “kill switches” to preserve unintended persistence. The aim is not passive observation but *principled* intervention—advancing human goals while respecting the integrity of microbial systems.

Perhaps the most urgent ethical frontier is healthcare's relationship with the holobiont self. The traditional war on microbes—exemplified by broad‐spectrum antibiotics—is increasingly unsustainable with the advent of antimicrobial resistance and microbiome disruption (Smith 2024). Emerging alternatives such as phage therapy (Sulakvelidze et al. 2001), faecal microbiota transplants (Weil and Hohmann 2015), and probiotic hospital surfaces (Hill et al. 2014) reframe patients as ecosystems. For example, surfaces inoculated with *Bacillus subtilis* have been shown to reduce pathogens such as *Staphylococcus aureus* and *Clostridium difficile* by 50%–89% compared to chemical disinfectants. Yet institutional adoption remains slow. Medical education still separates human physiology from microbiome science, and clinical trials rarely assess microbial impacts (Jones 2023). Reform would mean embedding microbiome impact assessments for new drugs or redesigning ICU environments that inhibit pathogens without relying on biocides (Vandini et al. 2014).

These cases—from material science to bacterial signalling to healthcare—are not exhaustive principles but serve as proof‐of‐concept for a broader paradigm. They show how collaboration can replace exploitation when microbes are considered as partners with stakes in the outcome. The lesson is not only technical but existential: in the face of climate change and pandemics, microbial wisdom—refined over billions of years—is not a resource to extract but a language to learn. The classroom is everywhere, from soil to skin, and the curriculum is human survival in a fundamentally microbial world (Haraway 2016).

## 10 Education as Negotiation: Learning From Microbial Pedagogies

Microbial pedagogies offer profound lessons—if we are willing to listen. At the deepest scale, microbes are our oldest teachers. Fossilised biofilms, dating back 3.8 billion years, reveal ancient microbial cities built on cooperation and resource‐sharing (Dodd et al. 2017). LUCA, the *Last Universal Common Ancestor*, likely a heat‐loving archaeon, encoded the metabolic logic shared by all life (Weiss et al. 2016). Through endosymbiosis, free‐living bacteria became mitochondria—the engines of our cells (Margulis 1970). We are not just descended from microbes; we are composed by them, our bodies ongoing negotiations with ancestral kin. This lineage challenges more than biology. In 1860, Darwin's claim of shared ancestry with apes caused public outrage (Desmond 1997). How much more unsettling is kinship with slime—sulphur‐breathing bacteria and collaborative biofilms?

Evolutionary theory reflects not just what we are, but how we wish to be seen. Resistance to microbial ancestry reveals a deeper anxiety: if we come from primordial ooze, what becomes of human exceptionalism? Yet these ancient organisms offer more than existential discomfort. Methanogens convert waste into energy (Tariq et al. 2025), microbial communities thrive through redundancy (Allison and Martiny 2008), and horizontal gene transfer favours connection over competition (Davis and Xi 2015). Metagenomics now lets us read these microbial *geostories*—a way of telling stories that includes not just humans, but also the Earth itself—from soil, seawater, and our own guts (Latour 2014; Handelsman 2004). But the challenge is ethical: why do we recoil from kinship with microbes while hosting their descendants within our cells? If we saw microbes as ancestors rather than invaders, how might we rethink medicine, agriculture, or education? The mycorrhizal networks beneath forests do not debate their kinship with trees—they act as kin. Perhaps the real lesson of deep time is not where we come from, but how we belong.

Microbes teach us the art of coexistence—not through neurons, but through electrochemical dialogues older than nervous systems themselves. They thrive as collectives, building biofilm cities with nutrient highways and waste‐recycling networks governed by quorum sensing: a molecular language, not a neural one. Humans, too, are holobionts—walking ecosystems whose bacterial inhabitants train our immunity, digest our food, and modulate our thoughts via the gut‐brain axis (Cryan and Dinan 2012). Coral reefs survive only through constant negotiation between animal, algae, and microbe (Forrest et al. 2023). Yet our moral and legal frameworks, rooted in neural exceptionalism, classify non‐neural intelligence as' primitive'—a taxonomic bias that justifies the exploitation of lifeforms with demonstrable capacities for cognition, cooperation, and adaptation. What does it mean that we privilege the biohack of neurons—a tool for memory and selective communication—as the sole benchmark for sentience, rights, or worth (Greene 2015)?

The bias is not just scientific; it is ideological. Speciesism (Singer 1974), like racism (Fredrickson 2002), draws arbitrary lines around moral worth. Just as 19th‐century audiences recoiled at kinship with apes, we now deny moral standing to beings without backbones, relegating microbes, plants, and invertebrates to the status of ‘automatons’. This hierarchy is self‐reinforcing: if only humans (or mammals, or vertebrates) can recognise moral claims, we assume only they can make them. But biofilms show that suffering and flourishing are not contingent on neurons. Bacteria starved of nutrients exhibit stress responses akin to pain (Fung et al. 2010); mycorrhizal networks redistribute resources to distressed trees (Simard 2018); even single‐celled organisms navigate trade‐offs between competition and cooperation (Leigh Jr. 2010). When we define sentience solely through neural complexity, we repeat the error of speciesism: mistaking a biological trait for a moral threshold.

The consequences of our cognitive biases are material. Industrial agriculture's degraded soils—stripped of microbial partnerships—mirror the dysbiosis in our own guts, both casualties of a worldview that values extraction over reciprocity. Laboratories experiment on octopuses, invertebrates with distributed cognition, and dismiss bee colonies, superorganisms with collective intelligence, because their suffering lacks the “dignity” of a spine. Legal systems grant corporations personhood while denying rivers or forests the right to exist unpoisoned. These are not oversights but symptoms of a paradigm that equates intelligence with centralisation and privileges lifeforms that resemble humans.

Tools like microfluidics and citizen science are forcing a reckoning by making microbial intelligence visible. If governance can be decentralised, as in biofilms, if memory can be epigenetic, as in bacteria, if decision‐making can be collective, as in mycorrhizal networks—then our ethical frameworks must also evolve. Recognising soil mycorrhizal networks as critical infrastructure would move conservation beyond anthropocentric goals. Legal recognition of microbial communities as “ecosystem engineers”, such as keystone fungi like *Rhizophagus irregularis*, could parallel protections for coral reefs and pollinators under biodiversity laws.

Policy could mandate agricultural impact assessments to evaluate mycorrhizal diversity loss, penalise fungicide overuse and reward farmers for maintaining microbial diversity through “soil microbiome credits,” verified through DNA metabarcoding. Remediation laws could require the fungal restoration using native inoculants, much like wetland recovery protocols. The precedent set by New Zealand's *Te Urewera Act*, which granted legal personhood to a forest, offers a model for extending protections to microbial life (New Zealand Government 2014).

The question is not whether microbes “think,” but why we refuse to notice. Designing policies, labs, and economies that respect microbial wisdom—embodied in their unique anatomy and behaviour—requires dismantling neural chauvinism—the unspoken belief that evolution's pinnacle is the human brain (Lahrach et al. 2024).

Coral reefs exemplify one of the most profound microbial lessons: survival depends on constant negotiation amongst species with no shared nervous system. Their fragility exposes the illusion of human autonomy; their resilience models an ethic of entanglement. If biofilms guided our moral compass, rights would stem not from synaptic complexity, but from relational vulnerability—the capacity to be harmed when connections are broken, and the agency to shape ecosystems without needing a brain. This transition does not diminish humanity but *repositions* us—not as apex thinkers, but as latecomers in a conversation billions of years old. Microbes do not claim personhood; they claim continuity. We are their legacy, and their fate is now ours to negotiate.

Microbes challenge the very foundations of perception. They navigate through chemotaxis, electrotaxis, and magnetotaxis—sensing gradients that we cannot perceive. Their memory is epigenetic, their lives unfold in minutes, and their intelligence is distributed across networks like mycorrhizal synapses between trees. To learn from them, we must shed sensory biases: treat soil scent as data, observe at microbial timescales, and use tools like atomic force microscopy to feel their world. With fluorescent biosensors and AI decoding quorum‐sensing dialects—we are only beginning to understand a language older than words.

This microbial curriculum reframes sustainability as an ancient practice, not a human invention. From LUCA's circular economies to biofilms' decentralised governance, microbes embody strategies refined over billions of years. Our task is not to mimic but to listen—designing with, not for, these primordial teachers. Unlearning the habits that gave rise to the Anthropocene begins by rejecting human exceptionalism and embracing microbial wisdom: distributed resilience, resource reciprocity, and co‐evolved symbiosis. Survival, microbes remind us, has never been a solo act—it is, and always has been, a collaborative practice.

## 11 Guidelines for Working With Microbial Teachers

Engaging microbes as mentors requires a fundamental change in both methodology and mindset. This means acknowledging their evolutionary knowledge and expanding our cognitive frameworks to accommodate non‐neural forms of problem‐solving. Microbial cognition operates through electron transfers, chemical gradients, and adaptive biochemical networks.

When *Geobacter* species respire using metals (Lovley 1993) or cyanobacteria adjust photosynthesis in response to light (Muramatsu and Hihara 2012), they exhibit context‐sensitive behaviours that challenge neural‐centric definitions of intelligence. While our interpretations of microbial communication will always be partial, this incompleteness is not a flaw—it's the space where mutual adaptation becomes possible.

Scientific rigour, in this context, means resisting anthropomorphism and designing experiments that prioritise observation and reciprocity over control. Like any dialogue, microbial engagement depends on the strength of our ‘arguments.’ Co‐culturing human intestinal cells with *Bifidobacterium* reveals cross‐kingdom signalling (Mirjam Rizzo et al. 2024), while kombucha fermentation—where *Saccharomyces* and *Acetobacter* negotiate shared resources (Lee et al. 2024)—offers a living model for circular, cooperative systems. These insights inform the *Implementation Framework for Microbial Mentorship*, bridging theory with practice across science, ethics, and policy (Table 1).

**TABLE 1:** Implementation framework for microbial mentorship: translating principles into practice.

Principle	Action	Example
Narrating organismal agency	Study extremophiles without anthropocentric assumptions (e.g., “survival instinct”)	*Deinococcus radiodurans* repairs DNA as inherent metabolism, not “resilience” (Cox et al. 2010; Cox and Battista 2005)
Dialogue with microbes	Citizen‐science tracking of urban biofilm adaptations	Public labs mapping *Pseudomonas* evolution in subway systems (Hester et al. 2018)
Ethics of receptivity	Mandate transparency in probiotic labelling (viability, strain specificity)	Laws requiring *Lactobacillus* supplement viability data (Hill et al. 2014)
Policy: Soil as commons	Soil microbiome credits for regenerative agriculture	Farmers paid for increasing mycorrhizal diversity in croplands (Jurburg et al. 2022)
SDG recognition	Formalise microbial contributions to UN SDGs (3, 6, 7, 9, 12–17)	SDG 15 (Life on Land) revised to include soil microbial carbon sequestration
Temporal negotiation	Align agricultural cycles with microbial lifespans (e.g., nitrogen fixers)	Rotational farming synced with Rhizobia activity periods (Liu et al. 2024)
Spatial cohabitation	Architecture designed to host air‐purifying microbiomes	Buildings with *Streptomyces*‐coated walls to degrade toxins (Andersson et al. 1997)
Microbial intelligence	Learn from bacterial “decision‐making” via quorum sensing	Swarm robotics programmed with *Vibrio fischeri* signalling logic (Cambier et al. 2020; Lupp and Ruby 2005)
Waste as metabolic dialogue	Redesign industrial byproducts as microbial food	Petrochemical waste metabolised by *Cupriavidus necator* into bioplastics (Jahn et al. 2024)
Justice for microbial labour	Legal protections for keystone species (e.g., gut/symbiont microbiomes)	*Bacteroides thetaiotaomicron* granted “ecosystem engineer” status in patents (Voigt and Taketani 2018)

*Note:* This table demonstrates how theoretical principles of microbial collaboration can be applied across scientific, ethical, and policy domains. Each row pairs a core concept (e.g., Narrating the organismal agency of microbes, Dialogue with microbes) with concrete actions and real‐world examples—from studying extremophiles without anthropomorphism to incentivising soil microbiome restoration through agricultural credits.

Ethical and policy frameworks must evolve in tandem. This means abandoning biocidal rhetoric (“war on germs”) in favour of stewardship: legally protecting keystone species like nitrogen‐fixing Rhizobia as ecological elders, and prioritising microbiome‐sparing alternatives to broad‐spectrum antibiotics—akin to clear‐cutting an old‐growth forest to remove weeds (Osterholm and Olshaker 2017). At institutional scales, microbiome‐informed design—such as self‐healing concrete embedded with *Sporosarcina pasteurii* —can cut construction emissions by 30% (Elgendy et al. 2024; Wang et al. 2014). Integrating microbial metrics into biodiversity assessments would also highlight peatland methanotrophs as climate allies, sequestering carbon more efficiently than many forests (Robinson et al. 2023). Ten of the 17 UN Sustainable Development Goals depend on microbes yet treat them as passive resources; recognising them as active stakeholders would reframe sustainability as a multispecies negotiation rather than a human managerial task (McDonald et al. 2024) (Table 2).

**TABLE 2:** Microbial contributions to sustainable development goals (SDGs): Key roles of microbial communities and their biomimetic lessons for global sustainability.

Sustainable development goal (SDG)		Role of microbes	What microbes can teach us
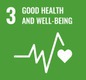	SDG 3: Good Health and Well‐being	Support the development of antibiotics, vaccines, and probiotics through biomedical applications	Offer insights into resilience, symbiosis, and the delicate balance between health and disease
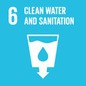	SDG 6: Clean Water and Sanitation	Drive natural purification by breaking down pollutants in wastewater and contributing to nutrient cycling	Demonstrate regenerative cycles and the power of decentralised, self‐sustaining systems
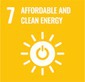	SDG 7: Affordable and Clean Energy	Enable the production of biofuels via photosynthetic organisms like microalgae and bacteria	Suggest alternative, bio‐based paths to energy autonomy and climate‐conscious technology
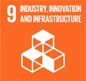	SDG 9: Industry, Innovation and Infrastructure	Form the basis of green biotechnologies, producing bioplastics, biofuels, and pharmaceuticals	Exemplify scalable innovation through minimal resource input and maximum adaptability
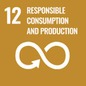	SDG 12: Responsible Consumption and Production	Transform waste into usable materials and support closed‐loop manufacturing (e.g., compostables, biosynthetics)	Embody circular resource management to inform innovation in the repurposing of by‐products into new resources
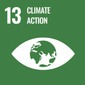	SDG 13: Climate Action	Sequester carbon dioxide through processes such as photosynthesis and methanotrophy	Teach carbon responsibility and microbial‐scale climate engineering potential
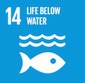	SDG 14: Life Below Water	Regulate marine nutrient cycles and sustain biodiversity through microbial ecosystems	Demonstrate resilience through interdependence and environmental stewardship in aquatic systems
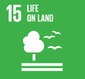	SDG 15: Life on Land	Maintain soil structure and fertility, supporting plant health and ecological balance	Highlight the importance of foundational infrastructure for terrestrial resilience
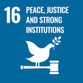	SDG 16: Peace, Justice and Strong Institutions	Indirectly promote peaceful development through shared microbial technologies and ethical frameworks	Offer (via biofilms and network logic) new economic and organisational models for cooperation, cohabitation, and shared ecological intelligence
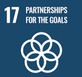	SDG 17: Partnerships for the Goals	Catalyse global collaboration through cross‐border research in microbial science and biotechnology	Demonstrate the value of shared knowledge and collective action across systems, species, and cultures via human trade, knowledge‐sharing, and microbial resilience

The lesson is clear: partnering with microbial teachers is not about adopting their strategies wholesale but creating conditions where their many years of innovation can inform—and transform—our fragile ecosystems. To illustrate this, Table 3 proposes microbial models for planetary redesign, highlighting microbial qualities that can enhance our knowledge, the models we can learn from, and the recommended actions we can take (Table 3).

## 12 Microbial Models for Planetary Redesign

**TABLE 3:** Microbial prototypes for post‐anthropocentric systems: Biomimetic strategies derived from microbial resilience, symbiosis, and resource diplomacy, with actionable insights for biotechnology and socioecological design.

Quality	Microbial model	Recommended action/insight
Resilience through redundancy	*Myxobacteria* form cooperative fruiting bodies under stress	Design decentralised systems that reorganise collectively during scarcity (e.g., distributed supply chains)
Symbiosis as infrastructure	Lichen (fungi + algae) merge into a single survival unit	Replace competitive frameworks with mandatory symbiosis (e.g., circular industrial ecosystems)
Regeneration as default	*Deinococcus radiodurans* repairs shattered DNA post‐radiation	Treat system collapse as a cyclical phase, not an endpoint (e.g., disaster recovery protocols)
Decentralised energy autonomy	*Geobacter* biofilms share electrons via nanowire grids	Build peer‐to‐peer energy networks that mimic microbial electron democracy
Waste as currency	Methanotrophs consume methane as food	Redefine waste streams as inputs (e.g., CO_2_‐to‐protein industrial loops)
Adaptive minimalism	Cyanobacteria thrive from deserts to oceans with minimal inputs	Innovate “low‐input, high‐output” systems (e.g., vertical farming modelled on algal efficiency)
Carbon diplomacy	Permafrost archaea regulate methane release via slow metabolisms	Synchronise human timelines with microbial rhythms and scales (e.g., long‐term carbon sequestration incentives)
Invisible aquatic stewardship	*Prochlorococcus* populations govern ocean oxygen silently	Value invisible ecosystem services (e.g., policy protections for microbial marine carbon pumps)
Mycorrhizal economics	Fungal networks trade nutrients between trees without central control	Replace extractive markets with reciprocal resource flows (e.g., soil carbon credits tied to fungal health)
Epigenetic policy	Bacteria share antibiotic resistance genes horizontally	Promote open‐source adaptation (e.g., rapid knowledge‐sharing platforms for climate solutions)

## 13 Conclusion: The Microbial University

The microbial turn in microbial literacy is not a metaphor but a survival imperative. Microbes are neither benign tutors nor a passive backdrop to human activity; they are formidable forces actively shaping Earth's systems, capable of symbiosis and devastation alike. The same biochemical ingenuity that allows *Geobacter* to purify water enables pathogens to outmanoeuvre antibiotics; the mycorrhizal networks that sustain forests can also contain invasive threats. Learning from microbes as ‘teachers’ requires neither romanticisation nor metaphorical reduction but adopts an ambitious practice of environmental diplomacy—one that recognises their agency while negotiating the terms of our shared planetary future.

As climate chaos accelerates, microbes offer more than knowledge; they demand a redefinition of human agency. Their classroom spans hospital wards and thawing permafrost, teaching through crises as much as collaboration. The COVID‐19 pandemic laid bare the costs of failed diplomacy, while cyanobacteria's oxygenation of the planet 2.4 billion years ago reminds us of their capacity for both creation and catastrophe. Our task is not to dominate or idealise these organisms, but to become adept translators of their language—aligning human needs with microbial metabolisms, as in bioremediation, while mitigating risks through ecological nuance, like balanced microbial interventions for oil‐spill clean‐up.

Engaging with microbial systems as pedagogical partners requires rethinking our knowledge frameworks, evaluation systems, and practice. This begins by recognising microbial agency—studying autonomous behaviours like bacterial quorum sensing, fungal network decision‐making, and viral recombination strategies—while upholding their “otherness”. Formalising this pedagogy requires temporal coexistence—for instance, aligning agricultural cycles like crop rotations with the metabolic rhythms of soil microbiome succession to optimise ecosystem health. It also demands spatial cohabitation, such as integrating architecturally embedded air‐purifying microbiomes into urban design to harness microbial metabolic functions for human benefit. Finally, it requires ethical frameworks that recognise keystone microbial species as critical stakeholders in environmental policy, ensuring their protection as foundational components of functioning ecosystems. These initial orienting recommendations and practices are the first phase of an ongoing interdisciplinary negotiation, establishing the conceptual groundwork for what must inevitably become a more expansive dialogue between human and microbial ways of knowing as this field develops.

This call to action is grounded in evolutionary fact. Over 3.8 billion years, microbes have engineered Earth's biosphere through biochemical innovation: oxygenating the atmosphere, regulating carbon and nitrogen cycles, and maintaining planetary homeostasis. Their evolutionary success exposes human exceptionalism as not only misguided but ecologically perilous. While we debate climate interventions, archaea have long managed methane fluxes; as we cultivate fragile monocultures, soil microbiomes sustain resilient, adaptive polycultures. The message is unambiguous: we are latecomers negotiating with life's original systems engineers. There are no second chances in this curriculum—each misuse of antibiotics, each disrupted microbiome, and each degraded wetland—is a failed systems test. Our continued viability depends on one critical capacity: the ability to engage in informed, adaptive negotiation with the microbial majority that maintains Earth's operating system. Microbes do not demand our recognition. They require that we learn to operate within their logic.

## Author Contributions

**Rachel Armstrong:** conceptualization, writing – original draft, funding acquisition, writing – review and editing, visualization, project administration, methodology.

## Conflicts of Interest

The author declares no conflicts of interest.

## Data Availability

Data sharing is not applicable to this article as no new data were created or analyzed in this study.
